# Mediterranean Diet on Sleep: A Health Alliance

**DOI:** 10.3390/nu14142998

**Published:** 2022-07-21

**Authors:** Egeria Scoditti, Maria Rosaria Tumolo, Sergio Garbarino

**Affiliations:** 1 National Research Council (CNR)—Institute of Clinical Physiology (IFC), 73100 Lecce, Italy; 2 Department of Biological and Environmental Sciences and Technology, University of Salento, 73100 Lecce, Italy; mariarosaria.tumolo@unisalento.it; 3 Department of Neuroscience, Rehabilitation, Ophthalmology, Genetics and Maternal/Child Sciences (DINOGMI), University of Genoa, 16132 Genoa, Italy; sgarbarino.neuro@gmail.com

**Keywords:** Mediterranean diet, sleep quality, sleep quantity, mental health, vasculoprotection, metabolism, inflammation, microbiota, melatonin

## Abstract

The Mediterranean diet is a plant-based, antioxidant-rich, unsaturated fat dietary pattern that has been consistently associated with lower rates of noncommunicable diseases and total mortality, so that it is considered one of the healthiest dietary patterns. Clinical trials and mechanistic studies have demonstrated that the Mediterranean diet and its peculiar foods and nutrients exert beneficial effects against inflammation, oxidative stress, dysmetabolism, vascular dysfunction, adiposity, senescence, cognitive decline, neurodegeneration, and tumorigenesis, thus preventing age-associated chronic diseases and improving wellbeing and health. Nocturnal sleep is an essential physiological function, whose alteration is associated with health outcomes and chronic diseases. Scientific evidence suggests that diet and sleep are related in a bidirectional relationship, and the understanding of this association is important given their role in disease prevention. In this review, we surveyed the literature concerning the current state of evidence from epidemiological studies on the impact of the Mediterranean diet on nighttime sleep quantity and quality. The available studies indicate that greater adherence to the Mediterranean diet is associated with adequate sleep duration and with several indicators of better sleep quality. Potential mechanisms mediating the effect of the Mediterranean diet and its foods and nutrients on sleep are described, and gap-in-knowledge and new research agenda to corroborate findings are discussed.

## 1. Introduction

Diet has become a cornerstone in the prevention and treatment of chronic noncommunicable diseases, with clinical areas of influence increasing over time as scientific evidence accumulates [1]. Epidemiological and clinical studies, along with mechanistic findings from cell and animal models, have indeed provided support for causal relationships between specific dietary patterns or foods/nutrients and health outcomes as well as disease development and progression, showing the ability of diet to significantly modify and often determine the lifelong health trajectories and chronic disease risk [2]. Accordingly, a comparative assessment of the disease burden attributable to diet in adult populations among 195 countries showed that suboptimal diets, i.e., diets high in sodium, low in whole grains, low in fruit, low in nuts and seeds, low in vegetables, and low in n-3 fatty acids, are the leading dietary risk factors for deaths and disability-adjusted life-years worldwide [3]. In contrast, diets characterized by increased consumption of vegetables, fruits, legumes, nuts, whole grains, unsaturated vegetable oils, fish, and lean meat or poultry (when meat was included) were associated with decreased risk of all-cause mortality among adults and older adults [4]. This has led to the inclusion of specific dietary recommendations into guidelines by public health entities and their prioritization in the health authority and research agenda to promote health and wellbeing, along with other modifiable lifestyle factors including smoking cessation and physical activity [5].

Among the healthiest diets, the Mediterranean diet is a plant-based dietary pattern that is increasingly become popular worldwide. The traditional Mediterranean diet is the dietary pattern consumed by the populations of the olive tree-growing areas of the Mediterranean basin before the mid-1960s. The traditional Mediterranean diet has entered the medical literature following publications of results from the Seven Countries Study, initiated in the late 1950s and showing that the Mediterranean diet is not simply, or mainly, a cholesterol-lowering diet, but exerts a range of beneficial health effects conferring longevity, better quality of life and preventing major chronic disease such as cardiovascular disease [6,7]. Other observational cohort studies, including the population-based European Prospective Investigation into Cancer and Nutrition (EPIC), confirmed the protection by the Mediterranean diet against chronic diseases, also including cancer. Randomized clinical trials, such as the PREDIMED (PREvencion con DIeta MEDiterranea) study and the Lyon Diet Heart Study, found impressive benefit of the Mediterranean diet in primary and secondary prevention of cardiovascular disease, respectively [6,7]. Later on, exponentially accumulating scientific evidence has consistently corroborated these findings and extended the health benefits of the Mediterranean dietary pattern against metabolic and neurodegenerative diseases, cognitive impairment as well as overall mortality, which is apparent among younger and older generations across Mediterranean and non-Mediterranean populations [7]. Concomitantly, many investigations have tried to identify the health promoting components(s) within the Mediterranean diet and the mechanisms mediating the beneficial biological effects. Evidence has shown the potential contributory role of some foods and nutrients herein present, such as olive oil, fish, fruits and vegetables, red wine, nuts, grains, and legumes [8]. Although the exact mechanism is not known, many interrelated and overlapping pathways have been hypothesized to play a role including: regulation of lipid and glucose metabolism; improvement of insulin sensitivity; protection against oxidative stress, inflammation, and platelet aggregation; enhancement of endothelial function; inhibition of tumorigenesis; and modulation of the gut microbiota [8].

Peculiar features of the traditional Mediterranean diet are: the abundance of olive oil (25–50 mL/day) as the main culinary fat for cooking and seasoning; the high consumption of vegetables (more than two servings per meal), fruits (one or two servings per meal), nuts (either as part of the recipes or as healthy snacks), cereals (one or two servings per meal), and legumes (more than two servings weekly); the moderate to high consumption of fish and shellfish (two or more servings weekly); the moderate consumption of poultry (two servings weekly), eggs (two to four servings weekly), dairy products (e.g., yogurt, low fat cheese, small portions daily), and alcohol, mainly wine, consumed preferably with meals (for women: ≤one drink/day; for men: one to two drinks/day); the low consumption of red meat and processed meats (less than one serving weekly); occasional consumption of foods rich in sugars and saturated fat (typical of Westernized dietary patterns); and use of herbs and spices as a key ingredient in the unique flavor of many Mediterranean dishes [9,10]. The richness in bioactive components such as vitamins, minerals, and phytochemicals (mostly polyphenols) from fresh fruits, vegetables, nuts, and legumes, contributes with synergistic actions to the health benefits of the Mediterranean diet. Though the intake of total fat is relatively high (30–40% of total energy intake), it mainly comes from virgin olive oil, tree nuts, and fatty fish, and therefore is predominantly unsaturated, mostly monounsaturated fatty acid (MUFA, more than 20% of total energy intake) and polyunsaturated fatty acids (PUFA), mainly n-3 fatty acids: consequently, the ratio of unsaturated to saturated fat is high. Furthermore, carbohydrates in the Mediterranean diet come mostly from unrefined, fiber-rich sources such as whole wheat and beans, while high quality proteins are provided by fish, sea foods, poultry, and legumes [9].

Taking into account adaptations to each country’s and region’s specific realities, the Mediterranean diet, recognized as Intangible Cultural Heritage of Humanity by UNESCO, is thought not only as a way of eating of the countries surrounding the Mediterranean Sea, but also as an integral part of a preserved social, cultural, and lifestyle sustainable model featured by moderation, biodiversity, local production, conviviality, culinary activities, regular physical activity, and adequate rest including nocturnal sleep and after-meal nap [9].

Besides diet, another essential health-promoting factor is sleep, an active physiological process necessary for life and normally occupying one third of our lives, playing a fundamental role for physical, mental, and emotional health [11]. Normal sleep architecture is comprised of nonrapid eye movement (NREM) sleep and REM sleep. NREM sleep is divided into three substages: stage N1, stage N2, and stage N3 (slow wave sleep). Older classification had four stages of NREM sleep. In the current rules, NREM stage 3 and NREM stage 4 are combined as stage N3. Sleep stages occur in cycles lasting 90 to 120 min each, with four to five cycles occurring during a typical night of sleep. Shifting of stages occurs over the course of the night, typically with an increased percentage of NREM sleep in the first half of the night and an increased percentage of REM sleep in the second half of the night [12]. Well recognized indicators of sleep quality are sleep latency, i.e., the length of time, in minutes, it takes to transition from wake to sleep (normal range for good sleep quality: 10–20 min), and sleep efficiency, i.e., the percentage of time in bed that is spent asleep (normal value for good sleep quality: above 85%).

Sleep patterns and sleep need are influenced by a complex interplay between genetic, behavioral, environmental, and social factors [13]. Expert consensus recommendations suggest that adults should obtain good sleep quality and duration (a minimum of 7 h per night) to promote optimal health and wellbeing [14,15]. Poor sleep quality and quantity can result from comorbid clinical conditions or sleep disorders, such as insomnia and sleep apnoea, but may also derive from the modern lifestyle with constant social, work, and family commitments, night- and shift-work, and the late-night use of technology (i.e., smartphones, computer, and television), which lead to circadian sleep–wake cycle disruption, chronic insufficient or poor quality sleep among adults as well as children and adolescents [16,17,18,19]. Serious consequences may arise from sleep curtailment ranging from fatigue, excessive daytime sleepiness, depressed mood, poor daytime functioning, and impaired cognitive and safety-related performance, to an increased risk of adverse health outcomes, including weight gain, obesity, type 2 diabetes, hypertension, cardiovascular, and neurodegenerative diseases, cancer as well as all-cause mortality [20,21,22,23]. Plausible biological mechanisms linking poor sleep and chronic disease risk involve endocrine, metabolic, and immune-inflammatory pathways [24], which are physiologically influenced by nocturnal sleep and whose dysfunctions play a determinant role in the development and progression of chronic diseases [25,26].

Sleep is therefore a modifiable risk factor for the development of chronic diseases, making it a target for intervention strategies [27,28]. Interestingly, in a prospective cohort study adding adequate sleep (≥7 h/night) to traditional healthy lifestyle factors, such as physical activity, a healthy diet (Mediterranean diet), moderate alcohol consumption, and nonsmoking, conferred further benefit against cardiovascular disease risk compared with no addition [29].

Besides being both key targetable lifestyle determinants of overall health and chronic disease risk, diet and sleep are linked by a bidirectional relationship. Indeed, qualitatively and/or quantitatively insufficient sleep or mistimed sleep may lead to overfeeding, metabolic impairments with weight gain and obesity, and poor diet quality, and, conversely, diet may influence sleep quality and duration so that nutritionally unbalanced diets or mistimed eating patterns negatively impact sleep parameters [30,31,32].

Accumulating scientific evidence from observational and clinical studies, as synthetized by recent literature reviews [33,34,35,36,37], suggests that dietary factors may impact on—and predict—sleep outcomes in otherwise healthy or clinical populations. In general, it has been shown that foods rich in melatonin (a sleep promoting hormone), or its precursors tryptophan and serotonin, micronutrients (vitamin D and B, magnesium, zinc), carbohydrate-containing foods, food items including cherries and fish, can improve sleep parameters (e.g., sleep latency, time, efficiency). Contrarily, caffeinated and sugar-rich beverages as well as high fat (mainly saturated fat) and processed foods may negatively affect sleep quality and duration [38,39].

However, nutrients and foods are not consumed in isolation but in combination within dietary patterns (as defined a priori through a validated score or defined a posteriori through data-driven methods) that are expected to more extensively and differentially impact on biological and behavioral processes and hence be more predictive of overall health status and disease risk than individual foods or nutrients [40]. Furthermore, a more sustainable lifestyle approach is thought to be via the modification of the dietary pattern as a whole, instead of simply supplementing or depleting some specific foods or nutrients. However, the influence of dietary patterns on sleep has been less explored [41,42,43,44], and most of the available evidence in this context has been focused on the effects of the Mediterranean diet on sleep and to see whether sleep improvements might be an additional health benefit of this diet.

The aim of the present scoping review is to synthetize and discuss scientific evidence on the effects of the Mediterranean diet on sleep outcomes (quality and duration), with a focus on potential plausible mechanisms underlying this association.

## 2. Literature Search Methods

**Figure 1:**
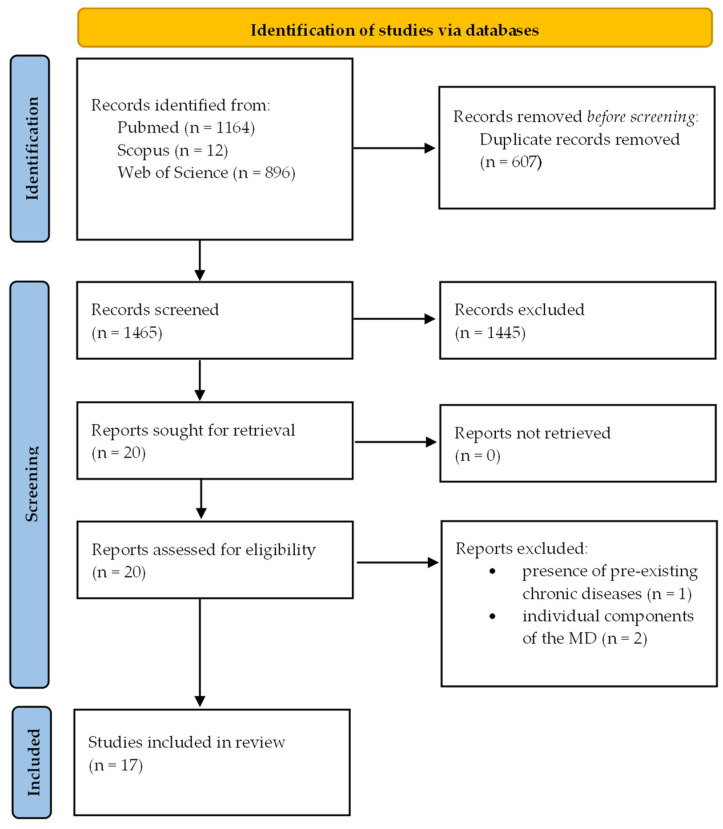
Flow diagram of the study selection process.

A literature search in PubMed, Scopus, and Web of Science was conducted from inception until April 2022, using the following key search terms, including MeSH terms, which were determined in accordance with the PICO (Population, Intervention, Comparison, and Outcome) method: “adolescents” and “adults” for population; “Mediterranean diet” and “Mediterranean dietary pattern” for intervention; and “sleep”, “sleep quality”, “sleep quantity”, and “sleep duration” for outcomes. Studies were included if conducted in otherwise healthy individuals, assessed sleep using questionnaire or polysomnography/actigraphy, and had cross-sectional, prospective/retrospective, or clinical intervention design. Studies were excluded if they: (1) were conducted in patients with preexisting chronic diseases; (2) did not have full text articles available in the English language; (3) investigated individual components of the Mediterranean diet rather than the whole diet as the exposure; or (4) examined multiple interventions/exposures, e.g., Mediterranean diet plus physical exercise or plus pharmaceuticals, or there were no arms to control for the effect of the combined treatment (Figure 1). The search strategy was supplemented by manually reviewing the reference list of all retrieved articles.

## 3. Main Findings

Following the literature search and articles selection, 17 studies were included in the present review [45,46,47,48,49,50,51,52,53,54,55,56,57,58,59,60,61]. A summary of the included studies and findings is reported in Table S1. Adherence to the Mediterranean diet was assessed using a priori derived scores, which are constructed through evidence from the scientific literature and encapsulate this dietary pattern into a numeric score for assessment of health outcomes [62]. For all scoring systems, a higher score reflects higher compliance to the traditional Mediterranean diet or the recommendations of the Mediterranean diet pyramid. Starting from the first Mediterranean diet score developed by Trichopoulou in 1995 and updated thereafter [63], other scoring systems (either modified or new) including the Mediterranean diet adherence score (MEDAS) [64], the Mediterranean diet adherence score based on the literature (MEDI-LITE) [65], and the alternate Mediterranean diet scale [47], have been developed and adapted to also take into account the cultural and geographical context of the specific populations studied, also in non-Mediterranean countries, thus often limiting interpretation and across-study comparisons. Sleep outcomes were generally self-reported using validated questionnaires, such as the Pittsburgh Sleep Quality Index (PSQI), but also using one or two questions not validated, asking about usual sleep duration, subjective sleep quality, or ease of getting to or maintaining sleep. Only in two studies were objective measures, such as actigraphy, used to assess sleep [48,57], while information on objective sleep architecture that can be derived using polysomnography is lacking.

All but one of the studies were observational, mainly with a cross-sectional design and only two studies with a longitudinal design, one in older adults [47] and another in adult women [61]. One of the main limitations is the paucity of intervention studies with the Mediterranean diet, thus not allowing to infer directionality or causality of the association. A randomized controlled feeding trial was conducted in overweight and obese adults to assess the short-term effect of two Mediterranean diet patterns with different amounts of lean unprocessed red meat (500 g/wk, which is a typical US intake, or 200 g/wk, as recommended in heart-healthy eating patterns) on several indicators of personal wellbeing, including sleep quality and sleep pattern [57]. The study did not find either any significant and robust effects of each Mediterranean diet pattern or a difference between the two patterns on sleep parameters, but the intervention duration was short (5 weeks) and the study not adequately powered [57].

Most studies were conducted in Mediterranean countries, including Spain, Greece, Italy, France, Iran, Jordan, but evidence also came from the USA and Northern Europe. Due to the small sample size or the inclusion criteria of the studies, the potential role of race/ethnicity, as well as postmenopausal women, which are at increased risk of poor sleep [13,66] in association with the Mediterranean diet with sleep, received little attention [48,61]. One study [50] found a positive association between the Mediterranean diet and sleep quality in pregnant women during the pregnancy course, where sleep disturbances are common and may be linked to metabolic anomalies as well as negative birth outcomes [67,68].

Although the adult population was the age group evaluated in the majority of the studies, in a few studies the effects on the Mediterranean diet were also assessed in adolescents [45,46,49,58], who are relevant to study being particularly vulnerable, mostly in recent years, to unhealthy behaviors such as sleep deprivation [17], lower physical activity, poor diet, and irregular eating patterns [69].

Despite the limited number of studies available, overall study results point to a beneficial impact of adherence to the Mediterranean diet pattern on sleep quality and sleep duration in both adolescents and adult individuals at cross-sectional and prospective evaluations. Specific features of sleep quality, such as sleep efficiency, sleep latency, daytime dysfunction due to sleepiness, and sleep disturbances resulted in improved greater adherence to the Mediterranean diet [51,54,61]. Furthermore, the presence of insomnia symptoms, a prevalent disordered pattern of sleep, was evaluated as an outcome in some studies [48,53,60]: here, in accordance with other findings in clinical populations [70], the Mediterranean diet was associated with a reduced risk of insomnia symptoms and of the most severe phenotype of insomnia in conjunction with short sleep duration, which is associated with worse cardiometabolic outcomes compared with insomnia without short sleep duration [71].

Interestingly, the only retrieved study reporting no association of the Mediterranean diet with sleep measures was conducted in Sweden in elderly men [59], where the Mediterranean diet adherence score was adapted to the dietary habits and food products of the Swedish population, and was assessed for association with only two sleep parameters, i.e., self-reported sleep initiation or maintenance problems.

In some studies, subgroups analyses showed that factors including age, gender, and body weight influenced the association between the Mediterranean diet and sleep. Indeed, in the MEAL study [51] the benefit of the Mediterranean diet on sleep quality (in particular sleep latency) was observed only in normal/overweight individuals (highest vs. lowest quartile of adherence score, OR = 2.30, 95% CI: 1.49, 3.54), but not in obese participants (highest vs. lowest quartile of adherence score, OR = 1.12, 95% CI: 0.33, 3.79). This result may be related to the beneficial modulation by the Mediterranean diet and its main components of metabolic profile, adiposity, and body weight status [72,73,74]. Therefore, the positive association between Mediterranean diet adherence and sleep quality may be mediated by improvement of weight status and obesity, which have a negative impact on sleep quality [55].

Furthermore, in the HELIAD study [54], when the analysis was stratified according to age, sleep quality was positively associated with Mediterranean diet adherence only in individuals aged ≤75 years (*p* < 0.001) and not in older (>75 years) individuals (*p* = 0.675), in which the presence of multicomorbidity and related polypharmacy and/or the increased occurrence of sleep problems could have masked or diluted the efficacy of the Mediterranean diet. In the French Three City Study [53], the inverse association between Mediterranean diet adherence and insomnia symptoms was significant only in women (OR = 0.80, 95% CI: 0.64−1.00) and not in men (OR = 0.93, 95% CI: 0.69−1.26), possibly due to hormonal or behavioral factors predisposing women to a higher prevalence of insomnia symptoms compared with men, and to a greater susceptibility to dietary influence. A similar gender difference in the effect of Mediterranean diet adherence on sleep has been observed in another study [52], where lower adherence to the Mediterranean diet was associated with shorter sleep duration in women, mainly linked to lower consumption of legumes, vegetables, and fruits. 

## 4. Potential Mechanisms Underlying the Mediterranean Diet Effects on Sleep

Exploring the mechanisms potentially mediating the beneficial effects of the Mediterranean diet on sleep parameters, several hypotheses have been raised. This dietary pattern has a healthy profile of fat, proteins, and carbohydrates and a peculiar richness in polyphenols and vitamins, mainly provided by the moderate to high intake of fruits, vegetables, nuts, olive oil, cereals, and fish. Mechanisms associated with these foods and nutrients and their possible combinations might explain the benefit of the Mediterranean diet on sleep. On the contrary, a high consumption of red meat, saturated fat, and sugar-rich foods and beverages that are eaten occasionally in the Mediterranean-style diet and characterize unhealthy diets, was associated with negative effects on sleep quality and quantity, and with insomnia symptoms [31,49,50,55,75,76].

Plant-based diets [41,75,77], such as the Mediterranean diet, have been shown to be associated with better sleep quality and/or duration. Indeed, though not consistent, evidence supports the association of intakes of fruits, vegetables, legumes, and their fiber and protein content, with improved sleep parameters [76,78]. These food items as key components of the Mediterranean diet have been found to contribute to the favorable epidemiological association between Mediterranean diet adherence and sleep in both longitudinal [61] and cross-sectional analyses [49,50,55,61]. Interestingly, in some studies [50,51,55] olive oil consumption, which is typical of the Mediterranean diet and a rich source of MUFA (55–83% olive oil fat) and polyphenols (50–800 mg/kg olive oil, about 2% of oil weight), have also emerged as potentially protective for sleep.

Regarding dietary fats, in accordance with cross-sectional findings [31], among major nutrients included in the Mediterranean diet, a higher intake of MUFA to PUFA ratio and of unsaturated fat, predicted better sleep quality at follow-up [61]. Although with mixed results [79], fatty fish (>150 g three times a week), a major food source of unsaturated fat, as well as plasma concentrations of n-3 fatty acids, including docosahexaenoic acid (DHA), and DHA supplementation were found to be positively associated with sleep quality in adults [55,75,80,81] and pediatric populations [82]. Confirmatory results have been reported in a very recent study in which higher plasma DHA concentrations were related to earlier sleep timing and longer sleep duration on the weekends in Mexican adolescents [83].

One of the most characteristic features and bioactive components of the Mediterranean diet is the richness in polyphenols, which are antioxidant compounds naturally present in plant foods and beverages (Table 1). They have gained increasing scientific attention due to their biological activities, their great abundance in human diet, and their role in the prevention of various chronic degenerative diseases, such as cancer, cardiovascular, and neurodegenerative diseases, through mechanisms also independent of their conventional antioxidant activities [84]. Over 500 different molecules having a polyphenol structure (i.e., several hydroxyl groups on aromatic rings) with different properties and bioavailabilities have been identified in edible plants, where they are synthetized as secondary metabolites for defense against biotic and abiotic stresses. They encompass five main groups according to structure: phenolic acids, flavonoids, stilbenes, lignans, and others. The flavonoid class is further divided into six subclasses including flavonols, flavones, isoflavones, flavanones, anthocyanidins, and flavanols [85]. In the European PREDIMED study, the mean total polyphenol intake was 820 ± 323 mg daily, mainly provided by fruits (44%), vegetables (12%), alcoholic (red wine) (6%) and nonalcoholic (coffee) beverages (55%), cereals (5%), olives and olive oil (11%), cocoa products, nuts, and legumes (each food group around 1–2%) [86].

After ingestion, phenolic compounds undergo several transformations in the gastrointestinal tract and also after absorption into the blood [87]. Additionally, a great proportion of nonabsorbed compounds reaches the colon where polyphenols are metabolized by the resident microflora, generating a different array of bioactive metabolites that are absorbed, further transformed, and released into the circulation [87]. Therefore, the bioactivities of ingested polyphenols depend mostly on their metabolites than on the native compounds.

Though experimental animal and in vitro studies have provided some potential mechanisms of sleep regulation by polyphenols (as described below), few human observational studies reported inconclusive results regarding the association of the polyphenol content of the diet and sleep parameters. A prospective study in UK women found that the total polyphenol content (but not polyphenol classes) of fruits and vegetables was inversely associated with sleep duration, in agreement with another study in Chinese adults reporting that soy isoflavone intake was associated with a low risk of long sleep duration [88]. On the contrary, adequate sleep duration and better sleep quality have been documented in association with high intakes of soy isoflavones in a study conducted in Japanese adults [89]. However, soy is not a typical Mediterranean food, and the effect on sleep of isoflavones from specific Mediterranean food items such as legumes has not been assessed. Recently, data from the Italian MEAL cohort study regarding the energy-adjusted total (poly)phenol intake estimated using the Phenol-Explorer database showed that a higher intake of some flavonoid subclasses (flavanones and flavones, mostly contained in fruits, vegetables, cereals, legumes, olive oil, and tea), phenolic acids, such as hydroxycinnamic acids (contained in fruits, vegetables, coffee, nuts, and cereals), and lignans (present in olive oil, cereals, nuts, legumes, fruits, and vegetables) were associated with a significantly lower likelihood of having inadequate sleep quality, only in normal weight individuals [90].

Human interventional studies also tested the effects of some polyphenol supplements on sleep but, again, the results were inconsistent, and many methodological limitations have been recognized including the small sample size, the health status of the participants, the use of supplements instead of food sources having different bioavailability, and the short duration of supplementation (as reviewed by [78]).

However, the combination of foods and nutrients in the frame of the Mediterranean diet pattern, rather than individual components, seems to better predict the sleep improvements associated with the Mediterranean diet in epidemiologic studies [51,55]. Further research, mostly clinical intervention and mechanistic studies are warranted to uncover efficacy by specific Mediterranean diet key components on sleep features.

Potential pathways involved in sleep regulation by the Mediterranean diet foods and nutrients are described below.

### 4.1. Metabolic and Vascular Improvements

Epidemiological and interventional studies have consistently provided strong evidence in support of Mediterranean diet benefits against metabolic and cardiovascular risk factors, thus preventing cardiometabolic diseases including obesity, type 2 diabetes, heart failure, coronary artery disease, cerebrovascular diseases, and peripheral artery diseases [91]. Improvements in adiposity and body weight, blood pressure, blood lipids, glucose metabolism and insulin sensitivity have been reported in association with adherence to the Mediterranean diet [72,92,93,94]. These effects may beneficially affect brain function, cognition, and mood [95,96,97], which are also important to sleep.

The control of body weight and body fat composition by the Mediterranean dietary pattern [72] and its key components including polyphenols [98] is hypothesized as an important mechanism mediating favorable effects on sleep quality and duration exerted by this diet. Obesity, an established independent risk factor for metabolic and cardiovascular diseases, is pathogenically associated with a proinflammatory state featured by elevated circulating levels and altered circadian pattern of inflammatory cytokines, which are implicated in the adverse obesity-associated cardiometabolic consequence [99] and are also sleep-regulating factors [24]. Excess body fat may cause obstructive sleep apnea (OSA), a major sleep disturbance associated with heightened risk of cardiovascular and metabolic diseases [100]. A weight loss intervention based on the Mediterranean diet, compared with a prudent diet, in combination with physical exercise in obese people with sleep apnea showed greater reduction of the apnoea-hypopnoea index (AHI) during REM sleep as well as waist circumference, an indicator of visceral adiposity, although it cannot be determined whether the improvement in sleep parameter was mediated by decreased adiposity [101]. The Mediterranean diet intervention also reduced the levels of inflammatory markers in individuals with obstructive sleep apnea [102]. A lower body mass index and waist circumference, and a higher adherence to the Mediterranean diet were found to be associated with better sleep quality in middle-aged adults [55]. Similarly, an increase in body mass index and fat mass as well as an unhealthy eating behavior (lower adherence to the Mediterranean diet) were associated with short sleep duration and poor sleep in an adolescent population [49]. In the study by Godos et al. [51], the association between higher adherence to the Mediterranean diet and better sleep quality was evident only in normal weight/overweight subjects and not in obese ones, suggesting that the beneficial metabolic effects of the Mediterranean diet may contribute to mediate its favorable influence on sleep. However, this hypothesis needs further investigation, and the control for these metabolic factors should be taken into consideration as potential mediating factors of the epidemiologic associations found.

Protective effects have been documented for the Mediterranean diet [103] and its main components [104,105] on the vascular function and in particular the vascular endothelium, which plays an important role in the modulation of vessel tone, tissue perfusion, dynamic permeability, hemostasis, immune response, and angiogenesis. As such, endothelial dysfunction as a result of several insults, e.g., hypertension, diabetes, dyslipidemia, smoking, as well as ageing, is recognized to contribute to pathophysiology of many disease states, such as cardiovascular, neurological, and neurodegenerative diseases [106,107,108,109]. The link between endothelial function and sleep is bidirectional, with sleep disturbances causing endothelial dysfunction as an antecedent to atherosclerosis and cerebro- and cardiovascular disease [110], and endothelial function being crucial for brain health and function, not only by contributing to the systemic vascular tone, immune function and hemostasis, but also by controlling, at the neurovascular unit, blood flow and blood–brain barrier function and interacting with surrounding brain tissue [111]. Furthermore, the recently discovered ability of the cerebral endothelium to contribute to the synthesis and secretion of the neurotrophin brain-derived neurotrophic factor (BDNF), a crucial mediator of synaptic plasticity and synaptic communication, which is implicated in neuronal survival, learning, memory, appetite, and sleep [112,113,114], makes any improvements of the endothelial function, as observed with the Mediterranean diet, an important contributor to preserving brain function and regulating sleep. Notably, plasma BDNF concentrations, which are lower in individuals with sleep disturbances [113], were improved by a Mediterranean diet intervention, mostly in depressed participants [115].

### 4.2. Blunting of Inflammation and Oxidative Stress

Chronic low-grade inflammation and an imbalance in the oxidant/antioxidant system are crucial pathogenic processes in the development and progression of chronic diseases such as diabetes, cardiovascular diseases, cancer [116], as well as mental and neurodegenerative diseases [117,118].

While low levels of cytokines regulate physiologic sleep, neuroinflammation, which is characterized by activation of microglia (the resident immune cells of the central nervous system), excessive release of proinflammatory cytokines, oxidative stress and neuronal damage, has been hypothesized to contribute to altered circadian rhythms and to poor sleep quality and quantity [24,119]. Several studies examined the associations between inflammatory markers and sleep health [120,121]. A better profile of circulating biomarkers of inflammation (C reactive protein [CRP]), oxidative stress (γ-glutamyl transferase [GGT]), and antioxidant capacities (bilirubin, carotenoids, uric acid, vitamins A, C, D, and E) has been associated with adequate sleep quality and duration [120,121]. Furthermore, these biomarkers may mediate the relationship between sleep and cardiometabolic health [122], with a particular role of oxidative stress and antioxidants for mediating the sleep duration–waist circumference and sleep duration–blood pressure relationships [122]. In support of the bidirectionality of the sleep-immunity linkage, sleep deprivation and/or inadequate sleep quality as observed in clinical populations and individuals with voluntary sleep curtailment or in experimental human and animal studies may lead to increased tissue and systemic levels of inflammatory markers and oxidative stress, that could mediate the associated adverse health outcomes [24]. The sleep–immunity/inflammation relationship raises relevant clinical implications of promoting sleep health by targeting inflammation and of improving or therapeutically controlling inflammatory response by targeting sleep.

The importance of the inflammatory and antioxidant effects of the diet on sleep has recently emerged from human cross-sectional studies. In one such study, the dietary inflammatory index (DII), a research tool based on literature evidence regarding the effects of diet on six inflammatory biomarkers (CRP, IL-1β, IL-4, IL-6, IL-10, and TNF-α) [123], was calculated based on the dietary intake data, and analyzed for correlation to sleep quality in a cohort of Italian adults [124]. Here, the highest category of DII (i.e., most proinflammatory) was associated with lower likelihood of having adequate sleep quality and in particular impaired sleep latency, and even more strongly after adjusting for the Mediterranean diet adherence underscoring that both DII and the Mediterranean diet were acting through a common denominator that may be inflammation [124]. Similar associations between higher DII scores and poor sleep quality [125] or daytime dysfunction [126] were observed in small cohorts of college students. In another study conducted in OSA patients the DII was positively associated with apnea severity and daytime sleepiness, and predicted rapid eye movement latency [127]. Data on about 30,000 individuals from the National Health and Nutrition Examination Survey (NHANES) found that adults consuming proinflammatory diets, as assessed by the DII, were more likely to present short sleep duration, long sleep duration, and/or self-reported sleep disturbances [128]. In a prospective cohort, changes over time in the DII toward an anti-inflammatory diet were associated with decreased wakening after sleep onset and improved sleep efficiency [129]. In a further in-depth analysis, a blood metabolomic study in participants from the Dietary Approaches to Stop Hypertension (DASH) trial showed that metabolites and pathways known to be implicated in inflammation and oxidative stress were associated with sleep variables (i.e., sleep midpoint, wake time) [130].

Regarding the effect of the antioxidant potential of diet on sleep, one study in postmenopausal women in Iran found that a higher dietary total antioxidant capacity (TAC), a tool for assessment of healthy effects of dietary antioxidants and estimated by measuring the oxygen radical absorbance capacity (ORAC) of selected foods, was associated with a reduction in sleep problems and other menopausal symptoms [131]. Another Iranian study in diabetic women confirmed that a higher dietary TAC, based on the ferric reducing ability of plasma (FRAP) and ORAC databases, was related with a lower risk of poor sleep and psychological disorders [132].

Though these data are only correlative in nature and cannot prove causality, they suggest that an improvement (or worsening) of inflammatory status and oxidative stress by diet could be a plausible mechanism for sleep modulation. A significant part of the health benefit and disease prevention ascribed to the Mediterranean diet has been explained by its antioxidant and anti-inflammatory properties. Indeed, observational and intervention studies [133,134,135] have shown that the Mediterranean diet is associated with increased serum TAC levels and antioxidant enzymes (superoxide dismutase and catalase) activities. Similarly, human studies have evaluated the impact of the Mediterranean diet on biomarkers of inflammation and immune activation showing that, compared with a Western diet or a prudent low-fat diet, the Mediterranean diet exerts an anti-inflammatory and immunomodulating effect through the downregulation of the levels of leukocyte and endothelial adhesion molecules, proinflammatory cytokines and their receptors, acute phase proteins (e.g., CRP), platelet and leukocyte counts, leukocyte trafficking, and chemoattractant molecules [103,136,137,138,139].

The Mediterranean dietary pattern is rich in foods, including olive oil, fruits, vegetables, nuts, fish, red wine, and of nutrients and nutrient combinations including vitamins, polyphenols, and unsaturated fatty acids, mainly MUFA and n-3 PUFA, which have shown to exert antioxidant [140,141] and anti-inflammatory effects [142,143,144,145,146] both at the systemic and cellular levels [147,148,149], in different cells and tissues including the brain, as discussed in the following paragraph. Interestingly, some of these foods (e.g., olive oil, fish, fruits) and nutrients (e.g., polyphenols, n-3 PUFA) have shown to be positively correlated to sleep quality in observational studies [51,61,90]. Therefore, it seems plausible that there is a contribution of the antioxidant and anti-inflammatory properties of the Mediterranean diet to the observed improvement of sleep, although causation cannot be proven by such findings and should be verified by interventional studies.

### 4.3. Neuroprotection

Observational data and limited evidence from clinical trials suggest that the Mediterranean dietary patterns improve cognitive performance and reduce the risk for cognitive decline and dementia, including Alzheimer’s disease [150,151,152]. Moreover, high adherence to the Mediterranean diet has been associate with a decreased risk of depressive disorders [153,154].

Besides indirect actions through peripheral effects (e.g., enhancement of endothelial function and cerebrovascular blood flow), actions inside the brain have been suggested as potential mechanisms. Indeed, the antioxidant, anti-inflammatory and vasculoprotective properties of the Mediterranean diet and its components have been hypothesized to contribute to prevent or dampen vascular dysfunction and neurodegenerative processes, and hence to improve brain function, cognition, and mood [152], which are all related to sleep [155,156,157]. Furthermore, direct neuroprotective effects have been documented for some Mediterranean diet nutrients, such as n-3 PUFA [158] and polyphenols [159].

Longer-chain n-3 fatty acids (eicosapentaenoic acid [EPA], DHA) are synthetized by shorter-chain n-3 fatty acids, such as alpha-linolenic acid (ALA) [160]. However, biological conversion is inefficient, especially during aging, and shorter-chain fatty acids cannot be synthesized by humans. Therefore, diet is the most important source of these fatty acids, mainly in the form of plant-derived ALA (abundant in green leafy vegetable, nuts, legumes) and fish- and marine-derived EPA and DHA, and their supplements [160,161]. The brain is particularly rich in n-3 PUFA, which incorporate into cell membranes and promote a favorable composition of phospholipids, influencing membrane fluidity and related function; as such, the maintenance of a balanced (low) n-6/n-3 PUFA ratio, which is part of the Mediterranean diet due to high dietary intake of n-3 PUFA and low dietary intake of n-6 PUFA, is crucial for brain development, normal neurological function, and in general for reducing the risk of chronic diseases [161,162,163]. Studies have shown that n-3 PUFA and their endogenous lipid metabolites (i.e., eicosanoids, lipoxins, resolvins, protectins, and maresins) have the ability to decrease [164] and/or resolve [165] neuroinflammation.

Besides this, by affecting gene and protein expression through the modulation of transcription factor pathways, and by positively regulating membrane-bound enzymes, signal transduction, ion channels, receptor activity and neurotransmitter binding, n-3 PUFA affect neurotransmission processes including dopaminergic, serotonergic, cholinergic, and glutamaergic systems [166,167,168,169,170,171]. Furthermore, animal studies showed that n-3 PUFA exert neuroprotective effects against cerebral ischemia, glial degeneration, neuronal apoptosis, and synaptic loss, and increased neurogenesis and synaptogenesis, as well as executive functions and learning abilities, thus preventing cognitive decline [172]. Concordantly, human observational studies [170,173] and some albeit not consistent intervention studies [174,175,176] have demonstrated neuroprotective action by high dietary intake of n-3 PUFA, in consonance with the protective effects against cognitive impairment and neurodegeneration exerted by the Mediterranean diet.

Antioxidant nutrients, including vitamins and polyphenols, represent other potential players in the neuroprotective effects of the Mediterranean diet [159]. Recent studies have demonstrated that polyphenols and their metabolites, mostly the less polar (lipophilic) ones, can cross the blood-brain barrier (BBB) and enter the brain at physiologically relevant concentrations, supporting their direct action in a neurological context [177,178,179,180]. Furthermore, the BBB endothelial cells have shown to further transform these metabolites into novel bioactive components [177].

In vitro and in vivo neuroprotective actions have been documented for several polyphenols and their metabolites, such as flavonoids, resveratrol, hydroxytyrosol and its derivatives, and hydroxycinnamic acids [181,182,183]. These include: cytoprotection of neurons, brain capillary endothelial cells, and astrocytes against insults such as oxidative stress, protein aggregates, and inflammatory stimuli [184,185,186,187]; reduction in glutamate excitotoxicity [181], and synaptic dysfunction [188,189]; blunting of microglia proinflammatory activation and cytokine production, interfering with mitogen-activated protein kinase (MAPK) and NF-κB pathways and promoting sirtuin 1 (SIRT1) pathway [177,188,189]; inhibition of reactive oxygen overproduction and oxidative stress via the upregulation of nuclear factor (erythroid-derived 2)-like 2 (Nrf-2) pathway and downregulation of pro-oxidant NADPH oxidase [188,190,191]; reduction in brain β amyloid pathology and tau protein aggregation, hallmarks of neurodegeneration [179,191,192]; increase in BDNF [193,194], and stimulation of adult neurogenesis [195]. Evidence also suggests that polyphenols can ameliorate synaptic plasticity [187,196]. These effects were accompanied in some animal and human studies by improvements in motor and/or cognitive parameters such as learning and memory [183,188,197].

With specific reference to sleep, animal studies have found that the hydroxycinnamic acid ferulic acid exerted dose-dependent sedative effects on locomotion activity and promoted sleep in mice by prolonging sleeping time and shortening sleep latency via a serotoninergic system-dependent mechanism [198]. The flavonoid hesperidin was shown to exert a sedative and sleep enhancing effect in mice, with a synergistic action when co-administered with gamma-aminobutyric acid receptor type A (GABA[A]) agonists including medications, such as benzodiazepines, widely used to treat insomnia and anxiety, as well as other flavonoids including apigenin [199]. This result opens up the possibility that flavonoids, besides being potentially valuable single drugs, may also be used with advantage in combination with benzodiazepines, thus achieving the same therapeutic effects with a substantial decrease in the benzodiazepine dose when used in synergistic combination with flavonoids. Apigenin [200] and (-)-epigallocatechin-3-O-gallate [201] have also shown to potentiate the pentobarbital-induced sleep in mice, possibly via chloride channel activation. Moreover, animal studies have demonstrated that dietary polyphenols may counteract the sleep deprivation-induced cognitive impairment, possibly through the inhibition of inflammation [202], or the activation of cAMP-response element-binding protein (CREB) and of mammalian target of rapamycin (mTOR) signaling pathways promoting synaptic plasticity [203,204]. These preclinical findings provide further research avenues for therapeutic exploitation of brain-targeting polyphenols to improve sleep and sleep disorders, and may contribute to the observed benefit of the Mediterranean diet on sleep features.

### 4.4. Melatonin Biosynthesis

A potential mechanism linking diet and sleep improvement involve the modulation of the tryptophan-serotonin-melatonin system. Serotonin and melatonin are two neurotransmitters involved in sleep regulation: in the central nervous system and in the gut the essential amino acid tryptophan is initially hydroxylated to 5-hydroxytryptophan, which is then decarboxylated with the formation of serotonin (5-hydroxytryptamine). With evening darkness, in the pineal gland of the brain serotonin is converted into melatonin (*N*-acetyl-5-methoxytryptamine), a neurohormone playing a central role in the sleep–wake cycle, sleep induction, and maintenance until dawn [205]. Melatonin supplements are widely used to treat insomnia and sleep apnea, helping in improving sleep quality and duration.

Diet may affect this biosynthetic pathway at different levels. First, some foods and beverages, most of which are typical of the Mediterranean diet, are natural sources of bioavailable melatonin precursors, i.e., tryptophan and serotonin, such as roots, leaves, fruits, seeds, dairy products, and fish, and of melatonin itself, including grapes, red wine, tomato, olive oil, purslane [206]. Accordingly, consumption of a Mediterranean dietary pattern was associated with increased tryptophan plasma concentrations, and these changes in tryptophan levels seemed to confer protection against cardiovascular disease incidence in high-risk individuals [207]. Interestingly, a metabolomic study has shown that the Mediterranean diet interventions, particularly when supplemented with extra virgin olive oil, modified the direct association between plasma metabolites deriving from tryptophan catabolism, i.e., kynurenines, and the risk of heart failure and atrial fibrillation, thus potentially counteracting the detrimental health effects of these metabolites [208]. This effect of the Mediterranean diet on the tryptophan–kynurenine pathway might have implication for sleep regulation, because kynurenines have been shown not only to play a role in peripheral and brain inflammation [209], but also to adversely impact sleep quality and cognition, at least in animal models [210].

Consuming tryptophan-enriched cereals for one-week increased plasma levels of serotonin and melatonin, increased sleep efficiency, and total sleep time, and decreased sleep latency and sleep fragmentation in elderly subjects with sleep difficulties, together with improvements in oxidative stress parameters and mood, in comparison with consumption of no cereals or control cereals [211]. A recent study, however, did not find any improvements in sleep quality in women with fibromyalgia consuming a tryptophan and magnesium-enriched Mediterranean diet, though a beneficial effect against anxiety symptoms, mood disturbance, eating disorders, and dissatisfaction with body image was observed [212]. Although more studies are needed to link the dietary intake of melatonin and its precursors in the frame of the Mediterranean diet to sleep parameters, the available evidence suggests a role for the diet content of tryptophan and their derivatives in the favorable modification of sleep by the Mediterranean diet.

A second pathway through which the Mediterranean diet can influence melatonin biosynthesis is by affecting the transport of tryptophan across the BBB and hence its bioavailability for melatonin synthesis. Of course, protein sources particularly rich in tryptophan may increase the plasma tryptophan and its transport into the brain. Of note, tryptophan enters the brain in a competitive manner with other large chain-neutral amino acids (LCNAA), so that dietary proteins rich in LCNAA may reduce tryptophan transport across BBB. Contrarily, by facilitating the peripheral uptake of LCNAA via the insulin response, a high carbohydrate diet favors tryptophan entry into the brain, thus promoting sleep through increased production of serotonin [213].

Finally, some nutrients characteristic of the Mediterranean dietary pattern, such as n-3 fatty acids (found in fish and nuts, seeds and dried fruit) and B-group vitamins (found in fruit and vegetables), affect melatonin biosynthesis in the brain by stimulating enzymatic reactions, including the conversion of 5-hydroxytryptophan into serotonin, which needs B6 vitamin, and the conversion of serotonin into melatonin which requires n-3 fatty acids [205]. Animal studies have shown that lower intake of DHA is directly related to lower concentrations of DHA in the pineal gland of the brain as well as with irregular melatonin release and the sleep–wake cycle [214]. Lower B vitamins intake has been related to later sleep timing in some studies [215]. Moreover, chronic treatment of aged rats with the stilbene resveratrol prevented the aging-associated reduction in serotonin levels in the pineal gland and other brain regions, indicating increased activities of limiting enzymes tryptophan hydroxylase, in concomitance with a restoration of impaired cognitive functions [216]. Similar results were observed with an antioxidant-rich diet [217]. Though sleep parameters were not measured in these studies, these findings propose some pathways that can mediate the influence of Mediterranean diet nutrients on brain health and sleep [218].

### 4.5. Microbiota Modulation

Another route for ingested nutrients to affect sleep physiology is the gut microbiota, a complex ecosystem located in the human gastrointestinal tract and able to exert a number of metabolic, immunologic, and neurobehavioral functions by interplaying with different tissues and organs [219]. There are many, important regulatory functions of the gut microbiota for the host so that gut dysbiosis, i.e., alteration of microbiota composition and/or functions, is linked to the pathogenesis of gastrointestinal, metabolic, vascular, neurological, and psychiatric disorders [219].

A bidirectional interaction exists between the gut microbiota and the brain, i.e., the microbiota–gut–brain axis, through various pathways including the vagus nerve, the immune/inflammatory system, the neuroendocrine system and bacterial metabolites, such as tryptophan, melatonin, serotonin, GABA, glutamate, norepinephrine, and short chain fatty acids (SCFA), thus influencing neurotransmission and behavior, including sleep [220].

Cell wall components of bacteria, such as lipopolysaccharide and fragments of peptidoglycans, are known to induce sleep via the stimulation of sleep-regulating cytokines [220]. However, products of live intestinal bacteria may also regulate sleep. Members of the *Firmicutes*, *Bacteroidetes*, and *Actinobacteria* phyla have been shown to produce sleep-regulating metabolites, such as GABA, glutamate, and serotonin [221]. SCFA (acetate, propionate, and butyrate, etc.), which are produced by the intestinal bacteria fermentation of nondigestible polysaccharides present in fibers, may also play a mediating role in sleep modulation by the intestinal microbiota, exerting their influence on brain function possibly through the vagus nerve, downregulation of hypothalamic–pituitary–adrenal (HPA) axis reactivity, anti-inflammatory effects and neurotransmitter regulation [222]. A recent study in rats found that intraportal injection and oral administration of butyrate increased NREM sleep [223].

Studies using germ-free mice have demonstrated the importance of commensal bacteria in regulating brain development, behaviors, and disease states of the central nervous system [224,225]. Furthermore, the gut microbiota composition, diversity, and function are crucial for the maintenance of normal sleep physiology, and sleep disturbance influences the gut microbiota [221,222,226]. Recent findings provide insights into the close, albeit still unknown, relation between gut microbiota and sleep. Studies in antibiotic-treated mice have found that disruption of gut microbiota leads to alteration of gut metabolites related to neurotransmission, with a depletion of serotonin and vitamin B6, a cofactor for dopamine and serotonin synthesis as well as for catecholamine metabolism [227]. Moreover, changes in sleep/wake patterns were observed in gut microbiota-depleted mice compared with normal control, with a shorter duration of NREMS episodes during the sleep phase and more frequent transitions between NREMS and REMS, suggesting alteration of circadian rhythmicity and fragmentation of NREMS episodes in the sleep phase [227].

Human observational studies also found that sleep quality positively correlated with the *Firmicutes*/*Bacteroidetes* ratio and microbial diversity [228]; in another study, using two metrics of a healthy gut ecosystem, i.e., the richness and diversity, Smith et al. [221] observed that microbiome diversity was positively associated with sleep efficiency and total sleep time, and was negatively associated with sleep fragmentation: in parallel, richness within the *Firmicutes* and *Bacteroidetes* phyla was associated with cognitive outcomes. Furthermore, gut microbiota composition in terms of the *Bacteroidetes* and *Firmicutes* phyla, *Lachnospiraceae*, *Rikenellaceae*, *Sutterellaceae*, *Prevotellaceae*, and *Pseudomonadaceae* families was also found to differ in habitual short sleepers compared with normal sleepers [226]. Interestingly, animal experiments with fecal microbiota transplantation, which may contribute to prove causality in the gut microbiota–sleep relationship, demonstrated that fecal microbiota from intermittent hypoxia-treated mice (a model of OSA) transferred to naive mice was capable of causing the appearance of somnolence in the recipient mice in the active phase [229]. In humans, fecal microbiota transplantation from healthy donors to patients with irritable bowel syndrome led to an increase in gut microbiota diversity along with improvements in subjective sleep quality and psychiatric symptoms (anxiety, depression) [230]. Overall, these results suggest the impact of gut microbiota on sleep health, and the potential role of gut microbiota dysbiosis and its modulation in influencing sleep disturbance.

Regarding the sleep-gut microbiota directionally, studies could not find a common gut bacterial signature associated with different sleep disorders, and results across studies are often inconsistent even within a specific sleep disorder. However, some evidence converges to the observation that disturbed sleep (e.g., sleep deprivation, sleep fragmentation, and OSA) is associated with changes in gut microbial composition and to dysbiosis, with a frequent observation of an increase in *Firmicutes* to *Bacteroidetes* phyla ratio or a reduction in gut microbiota diversity and richness, at least in some types of sleep disturbance. These microbiota alterations have been recognized to adversely affect the intestinal epithelial barrier and to lead to local and systemic inflammation and negative health outcomes [222].

Diet plays a major role in shaping the gut microbiota and, in particular, the Mediterranean diet has been shown to beneficially affect the abundance, composition, and metabolic activity of the gut microflora [231,232]. A seminal observational study showed that high adherence to the Mediterranean diet was associated with increased fecal SCFA levels, likely due to the higher proportion of *Prevotella* among *Bacteroidetes* and *Lachnospira* among *Firmicutes*, which are able to degrade carbohydrates not digestible by the host [233]. Contrarily, lower adherence to the Mediterranean diet, with increased consumption of animal protein, saturated fats, and simple sugars, was associated with higher urinary levels of trimethylamine oxide (TMAO) [233], a gut microbiota metabolite mostly deriving from animal proteins, and recognized as a potential risk factor for cardiovascular disease as well as for brain aging and cognitive impairment [234,235].

Compared with a Western diet, a 2-week intervention with a Mediterranean diet was associated with a higher abundance of butyrate-producing bacteria and a remarkable change in the metabolic activity of gut microbiota [236]. The ability of the Mediterranean diet to restore the gut microbiome dysbiosis has been also demonstrated in a randomized clinical trial in which obese subjects adhering to the Mediterranean diet for two years presented an improved insulin sensitivity and a concomitant increase in *Bacteroides*, *Prevotella*, and *Faecalibacterium* genera, and, most importantly, of the *Roseburia* and *Ruminococcus* genera and *Parabacteroides distasonis* and *Faecalibacterium prausnitzii* bacterial species, known for their saccharolytic activity and the ability to convert carbohydrates in SCFA [237]. Features of the Mediterranean diet that can be associated with these positive effects on the gut bacteria ecosystem include the high content in unsaturated fat, such as MUFA and n-3 PUFA, which have been shown to increase the abundance of beneficial bacteria including *Akkermansia* and *Bifidobacterium* and to reduce detrimental bacteria such as *Streptococcus* and *Escherichia* spp. [238]. The high fiber content of the Mediterranean diet also plays an important role, with a known prebiotic effect and being key bacteria fermentable substrates to generate SCFAs, increasing the abundance of *Bifidobacterium* and reducing the ratio of *Firmicutes*/*Bacteroidetes*, and promoting the growth and diversity of the gut microbiota taxa in humans and experimental animals [238].

Besides macronutrients, polyphenols of the Mediterranean diet are important modulators of gut bacteria ecosystems, thus influencing the gut microbiota–brain axis. On the one hand, ingested polyphenols can be utilized by gut bacteria and transformed into phenolic metabolites with increased bioavailability and bioactivity compared with the parent compounds [239]. These metabolites can exert health effects locally in the intestine and systemically on target organs. The gut microbiota composition and metabolic activity, which may depend on several external and host factors, may therefore influence the bioavailability and biological effects of polyphenols. Obesity can promote overgrowth of pathogenic microorganisms and is associated with perturbations in the composition and metabolic function of the gut microbiota, with some studies reporting an increased *Firmicutes* to *Bacteroidetes* ratio in obese subjects compared with normal-weight controls [240], which may impinge on the biotransformation of polyphenols and hence on their bioactivity [178]. On the other hand, ingested polyphenols, including resveratrol, quercetin, hesperidin, tannins, catechins, phenolic acids, and secoiridoids, can shape the gut microbiota by exerting a direct antimicrobial effect, selectively inhibiting the development of potential pathogenic species, such as LPS producers (*Escherichia coli* and *Enterobacter cloacae*) [241]; they also exert a direct stimulatory/modulatory effect on gut bacteria favoring an increased abundance of bacteria with health benefits to the host, as manifested, for example, by decreased *Firmicutes* to *Bacteroidetes* ratio, and an increase in *Akkermansia muciniphila*, *Bacteroides thetaiotaomicrom*, *Faecalibacterium prausnitzii*, *Bifidobacteria*, and *Lactobacilli*, thus modifying gut-derived metabolite profile, promoting a higher production of SCFA and serotonin, with parallel improvements of oxidative stress, inflammation, as well as metabolic, neurologic, and cognitive functions [239,242,243]. Small clinical trials and animal studies have found that olive oil and its main phenolic compounds favorably changed gut microbiota composition and metabolic function, so that an increase in *Bacteroidetes* or a reduction in *Firmicutes*/*Bacteroidetes*, and an increase in beneficial bacteria such as *Bifidobacteria*, and *Lactobacillus*, have been observed after olive oil polyphenol administration, in association with an increase in SCFA production [239]. A similar beneficial modulation of gut microbiota has been shown for red grape and red wine, or some vegetables [244].

Whether the sleep effects of the Mediterranean diet and its main components are mediated by the modulation of the gut microbiota, though plausible, remains unexplained and deserves further research in experimental animals and in humans.

## 5. Future Perspective and Conclusions

The epidemiological evidence gathered in the present review concordantly suggests a positive influence of the Mediterranean diet on sleep pattern, improving sleep quality and duration in otherwise healthy subjects. The underlying mechanisms are still partially explained and generally supposed, based on the biological plausibility of the multiple beneficial effects—and underlying mechanisms—exerted by this dietary pattern on (patho)physiological processes, including inflammation, oxidative stress, metabolism, neuroendocrine system, or cell signaling. Considering the role of physiological sleep in health maintenance and, contrarily, of sleep curtailment in chronic disease development, beneficial sleep modulation by the Mediterranean diet seems to represent an additional pathway through which the Mediterranean diet may prevent the risk for major diseases, including cardiometabolic, neurodegenerative and psychiatric diseases, and cancer.

Similarly to other health parameters modulated by the Mediterranean diet [245], though the Mediterranean diet is a rich source of potential sleep-promoting foods and nutrients, no single unifying factor exists in the relation between the Mediterranean diet and sleep, but the additive and synergistic actions of the multiple and pleiotropic nutrients combined in this dietary pattern seems to be more important than the effect of individual nutrients in providing the final effects on sleep. The whole diet reflects real life conditions where foods or nutrients are eaten in combinations and interact among them, so that it can be difficult to disentangle their independent effects. This comprehensive approach is emphasized by dietary guidelines and has been used in several clinical settings, including cardiovascular disease, cancer, and type 2 diabetes [246].

A bidirectional direct relationship links diet and sleep, with poor and/or short sleep negatively influencing dietary intakes, and in turn unhealthy diet unfavorably affecting sleep quantity and/or quality. Thus, a possible (vicious or virtuous) cycle can emerge between diet and sleep, which are both modifiable determinants of health outcomes. As such, the directionality of the associations found in the available epidemiological studies cannot be established.

As outlined in the earlier observations made by Ancel Keys in the Seven Countries’ Study and in the recently updated Mediterranean diet concept [9], the Mediterranean diet is a part of a healthy lifestyle pattern, which includes preference for seasonal, local, fresh and raw foods, eating with moderation and frugality, conviviality and social interactions, lengthy meals and post-lunch naps, leisure activities such as moderate physical activity, adequate and regular nocturnal sleep according to the circadian sleep–wake rhythm, and a less stressful way of life [245]. This implies that the health benefit against the risk of chronic diseases associated with adherence to the Mediterranean diet, as observed in epidemiological studies including those presented here, may depend on the clustering of healthy lifestyle features, which also include a better sleep.

Although many non-Mediterranean countries are increasingly adopting the Mediterranean dietary pattern due to its evidence-based international recognition as one of the healthiest dietary patterns, there has been a progressive worldwide abandonment of the traditional Mediterranean-style diet mostly in countries of the Mediterranean region [247]. This trend highlights the need of individual and public health policies prioritizing the preservation and promotion of the Mediterranean diet approach and lifestyle habits to counteract risk factors, including impaired sleep, and the increasing rates of chronic disease, mostly in times of economic crisis and pandemic outbreak. In parallel, a progressive nocturnal sleep curtailment has been documented over time, thus exposing individuals starting from early ages to the negative health consequences of poor or short sleep [16].

As an integral part of homeostasis, essential physiological functions, such as sleep–wake cycle, metabolism, immune system, and cardiorespiratory functions, are controlled by the circadian clock system, which coordinates internal time with the external environment resulting in 24-h day/night cycles. Disorders of the circadian rhythms have severe consequences on human health. In the modern 24/7 society, the school and work schedules and other social demands, the exposure to artificial light at night and the use of electronic devices can cause delayed sleep onset, poor sleep quality and sleep deprivation, which people attempt to compensate for during weekends, resulting in a misalignment between the internal circadian clock and the actual sleep–wake cycle, described as social jet lag [248]. This phenomenon is mostly pronounced among individuals who prefer late bedtimes and later awakening (evening chronotypes), and is associated with an increased risk of obesity and metabolic disorders. A recently discovered feature of social jet lag is its association with a lower adherence to the Mediterranean diet and irregular eating timing, which may be linked to sleep derangements and may contribute to explain part of the negative metabolic effects of social jet lag [249]. A similar circadian misalignment with poor sleep as well as poor food choices and irregular meals [250] can be observed in night- and shift-workers, which are frequently occurring in the current 24-h economy and are at an increased risk of chronic diseases. Dietary interventions based on Mediterranean diet might represent an effective strategy to counteract the detrimental effects of this work schedule, but studies are warranted in this context.

The following tasks might be implemented in future research and policy agenda:

- large prospective cohort studies are needed to assess the preventive effect of the Mediterranean diet on sleep and identify the potential qualitative and quantitative contribution of typical foods and nutrients;
- clinical intervention studies with the Mediterranean diet as exposure and sleep as outcome in large samples are needed to confirm associations and provide causality;
- preclinical studies in animal models could provide insights into mechanisms and pathways mediating the benefit of the Mediterranean diet on sleep features;
- objective neurophysiological tools for sleep assessment (actigraphy, polysomnography) should be used in the scientific studies;
- the effects of meal timing and frequency, and the influence of individuals’ chronotype in the relation of the Mediterranean diet and sleep should be investigated;
- more studies on both genders and in different age groups, including during pregnancy, are needed to substantiate the influence of the Mediterranean diet on sleep as a life-long exposome;
- the influence of risk factors, such as overweight/obesity, on the relation between the Mediterranean diet and sleep requires investigation;
- the role of gut microbiota in the Mediterranean diet–brain–sleep axis should be further studied, using strategies such as fecal microbiota transplantation, in order to assess whether and how the modulation of gut bacteria ecology by the Mediterranean diet could mediate the effects on sleep, potentially providing new therapeutic pathways and biomarkers.
- public health policy and health promotion programs should include focused attention on diet, especially the Mediterranean diet, and sleep.
- knowledge and eventually education about the diet–sleep relation should be improved among healthcare professionals including nutrition professionals, in order to implement the discussion of this topic with patients during routine healthcare practices in promoting the adoption of healthy and protective lifestyles.

These new directions for future actions would contribute to substantiate the clinical relevance of the Mediterranean diet for promoting adequate sleep and preventing sleep disturbances, ultimately leading to evidence-based dietary recommendations focused on Mediterranean-style diet to adopt early in life as part of a healthy lifestyle.

In conclusion, taking account of the limitations of the available evidence and pending future research, the Mediterranean diet can be considered an easy-to-implement and safe lifestyle intervention to promote proper sleep hygiene, favorably nourishing the connection between these two allies along the path towards wellbeing and health.

## Figures and Tables

**Table 1:** Main Mediterranean diet polyphenols, food sources and dietary intake.

Class	Subclass	Main Representatives	Main Food Source	Intake (mg/day)
Flavonoids	Flavanols	Catechin Epicatechin Epigallocatechin	Apples, red wine, tea, peaches, cocoa products, beans	26.7 ± 19.6
	Flavonols	Quercetin Kaempferol Myricetin	Spinach, beans, onions, lettuce	80.4 ± 32.7
	Flavanones	Hesperidin and its aglycone hesperetin Naringenin Didymin	Oranges, orange juice, red wine, tomatoes	132 ± 125
	Flavones	Apigenin Luteolin	Oranges, whole-grain wheat-flour bread, refined-grain wheat-flour bread	41.6 ± 26.1
	Isoflavones	Genistein Daidzen Glycitein	Beans, beer	0.003 ± 0.003
	Anthocyanins	Malvidin Cyanidin Delphinidin	Cherries, red wine, olives, strawberries	38.5 ± 37.4
	Phenolic alcohol and secoiridoids	Hydroxytyrosol, tyrosol Oleuropein Oleacein Oleocanthal	Olive oil	39.46 ± 29.37
Non-flavonoids	Stilbenes	Resveratrol	Red wine, white wine, grapes, strawberries	1.84 ± 3.39
	Phenolic acids	Hydroxycinnamic acids (cinnamic, p-coumaric, ferulic, caffeic, chlorogenic, and rosmarinic acids, verbascoside)	Coffee, potatoes, apples, olives	276 ± 146
		
		Hydroxybenzoic acids (p-hydroxybenzoic, gallic, syringic, protocatechuic, and vanilic acids)	Olives, red wine, walnuts, beer	19.1 ± 16.8
	Lignans	Secoisolariciresinol Pinoresinol 1-Acetoxypinoresinol	Olive oil, whole-grain wheat-flour bread	0.85 ± 0.36
	Tannins	Condensed tannins or proanthocyanidins (oligomers or polymers of flavanols) Hydrolyzable tannins or gallotannins, ellagitannin	Red wine, apples, peaches, plums, orange, green beans, lentils	117 ± 81

Data on polyphenol content were adapted from [86]. Food sources are reported in decreasing order of specific polyphenols content.

## Data Availability

No new data were created or analyzed in this study. Data sharing is not applicable to this article.

## Supplementary Materials

The following is available online at https://www.mdpi.com/article/10.3390/nu14142998/s1, Table S1: Characteristics and main results of included studies (by publication date).
