# Supplementary material for: Mediterranean Diet on Sleep: A Health Alliance
Source: Nutrients. 2022 Jul 21;14(14):2998. doi: 10.3390/nu14142998 (PMC9318336; doi:10.3390/nu14142998)
Supplement: Supplementary file 1 [file nutrients-14-02998-s001.zip › nutrients-1833477-supplementary.pdf]

Table S1. Characteristics and main results of included studies (by publication date).

Country; Study design	Participants (gender); Age (mean or range)	Mediterranean diet assessment tool/Interventions (duration)	Sleep outcome(s)	Sleep assessment tool	Main results	Reference
France; cross-sectional (Three City Study)	5,886 (MF); ≥ 65 yrs	11-item questionnaire	Insomnia symptoms (difficulty in initiating sleep, difficulty in maintaining sleep, and early morning awakening).	Self-report questionnaire	Only in female, Mediterranean diet adherence (i.e. reporting more than seven components) reduced the risk of insomnia symptoms.	Jaussent et al. 2011 [1]
Italy; cross-sectional	1,586 (MF); 12 ± 0.7 yrs	KIDMED	Sleep pattern (sleep duration, daytime sleepiness).	Self-report questionnaire for sleep duration and PDSS for daytime sleepiness	Adherence to the Mediterranean diet was positively associated with total and weekdays sleep time, and inversely with daytime sleepiness.	Ferranti et al, 2016 [2]
Spain; prospective with 2.8 yrs-follow-up (Seniors-ENRICA cohort)	1,596 (MF); ≥ 60 yrs	MEDAS	Sleep duration and quality (poor general sleep quality, difficulty falling asleep, awakening during the night, early awakening with difficulty of getting back to sleep, need to sleep at daytime, not feeling rested in the morning, use of sleeping medication, snoring and daytime sleepiness).	Self-report questionnaire	Mediterranean diet adherence was associated with lower risk of large (>2 h/night) changes in sleep duration and of poor sleep quality.	Campanini et al. 2017 [3]
Spain; cross-sectional	329 in the north and 284 in the south of Spain; 11-12 yrs	KIDMED	Sleep duration	Self-report number of hours of nightly sleep	Greater adherence to the Mediterranean diet was associated with higher sleep duration in northern children.	Arriscado et al, 2017 [4]
United States; cross-sectional (Multi-Ethnic Study of Atherosclerosis [MESA] Study)	2,261 (MF); 45–84 yrs	aMED	Insomnia symptoms (difficulty falling asleep, awakening during the night, early awakening, difficulty of getting back to sleep, sleep quality) and sleep duration.	WHIIRS for insomnia symptoms and 7-day actigraphy for sleep duration	Participants with moderate-high Mediterranean diet adherence score were more likely to sleep 6–7 h/night (vs. <6 h/night) and less likely to report insomnia symptoms in conjunction with short sleep duration.	Castro-Diehl et al. 2018 [5]

Greece; cross-sectional (Hellenic Longitudinal Investigation of Aging and Diet [HELIAD] study)	1,639 (MF); ≥ 65 yrs	11-item Mediterranean Diet Score	Sleep quality (sleep disturbance, sleep latency, somnolence, difficulty falling asleep, awakening during the night with difficulty of getting back to sleep, snoring, and awakening short of breath or with a headache) and sleep duration.	Medical Outcomes Study Sleep Scale and Sleep Index II	Adherence to the Mediterranean diet was positively associated with sleep quality but not sleep duration, only in individuals aged ≤ 75 yrs.	Mamalaki et al, 2018 [6]
United States; randomized controlled crossover feeding trial	41 (MF); 46 ± 2 yrs	MEDAS/Mediterranean diets with ~500 g/wk or ~200 g/wk of lean, unprocessed beef or pork (5 weeks)	Sleep quality (self-rated sleep quality, sleep latency, sleep duration, habitual sleep efficiency, sleep disturbance, use of sleeping medications, and daytime dysfunction) and sleep patterns (time spent in bed, time spent sleeping, sleep efficacy, onset latency, number of minutes awake after sleep onset.	PSQI and actigraphy	Independent of red meat intake, Mediterranean diet interventions did not change sleep quality and sleep pattern.	O'Connor et al, 2018 [7]
Italy; cross-sectional (Mediterranean healthy Eating, Aging, and Lifestyles [MEAL] study)	2,044 (MF); ≥ 18 yrs	MEDI-LITE	Sleep quality (self-rated sleep quality, sleep latency, sleep duration, habitual sleep efficiency, sleep disturbance, use of sleeping medications, and daytime dysfunction).	PSQI	In normal/overweight individuals, adherence to the Mediterranean diet was positively associated with overall sleep quality, including sleep duration.	Godos et al, 2019 [8]
Spain; cross-sectional (Deporte, ADOlescencia y Salud [DADOS] study)	269 (MF); 13.9 ± 0.3 yrs	KIDMED	Sleep quality (self-rated sleep quality, sleep latency, sleep duration, sleep efficiency, sleep disturbance, use of sleep medications, and daytime dysfunction) and sleep duration.	PSQI for sleep quality and wrist-worn accelerometer for sleep duration	Adherence to the Mediterranean diet was positively associated with sleep quality. Sleep quality was a mediator for the association between the Mediterranean diet and academic performance.	Adelantado-Renau et al, 2019 [9]
Sweden; cross-sectional (Uppsala Longitudinal Study of Adult Men [ULSAM] study)	970 (M); 71 ± 1 yrs	8-item questionnaire	Sleep quality (sleep initiation problems, sleep maintenance problems).	Self-report questionnaire	Adherence to the Mediterranean diet was not associated with sleep parameters.	van Egmond et al, 2019 [10]

Italy; cross-sectional (Obesity, Programs of Nutrition, Education, Research and Assessment of the best treatment [OPERA] study)	172 (MF); 51.8 ± 15.7 yrs	MEDAS	Sleep quality	PSQI	Adherence to the Mediterranean diet was positively associated with overall sleep quality.	Muscogiuri et al, 2020 [11]
USA; prospective with 1-y follow-up (American Heart Association Go Red for Women Strategically Focused Research Network study)	432 (F); 20–76 yrs	aMED	Sleep quality (self-rated sleep quality, sleep latency, sleep duration, habitual sleep efficiency, sleep disturbance, use of sleeping medications, and daytime dysfunction).	PSQI	Greater adherence to the Mediterranean diet at baseline was associated with better overall sleep quality and, in particular, with lower sleep disturbances and higher sleep efficiency at 1-y follow-up.	Zuraikat et al, 2020 [12]
Spain; cross-sectional (GESTAFIT project)	150 (F); 32.9 ± 4.6 yrs	Mediterranean Food Pattern score	Sleep quality (self-rated sleep quality, sleep latency, sleep duration, sleep efficiency, sleep disturbances, use of sleep medication, and daytime dysfunction).	PSQI	In pregnant women, adherence to the Mediterranean diet was positively associated with sleep quality during both the 16th and 34th gestational weeks.	Flor-Alemany et al, 2020 [13]
Italy; cross-sectional	409 (MF); 12.5 ± 0.6 yrs	KIDMED	Sleep duration and daytime sleepiness (indicative also for sleep quality)	Sleep duration (weekday sleep duration x 5 + weekend sleep duration x 2)/7); PDSS for daytime sleepiness and sleep quality.	Adherence to the Mediterranean diet was positively associated with sleep duration, sleep quality and lower daytime sleepiness.	Rosi et al, 2020 [14]
Jordan; cross-sectional	917 (F); 36.3 ± 10.3	PREDIMED score	Insomnia symptoms	Athens Insomnia Scale	Adherence to the Mediterranean diet was positively associated with better sleep quality and reduced insomnia symptoms.	Zaidalkilani et al, 2021 [15]
Costa Rica (Costa Rica Heart Study); cross-sectional	2169 (MF); 50-60 yrs	Modified aMED	Sleep duration	Self-report number of hours of sleep on weekdays and weekends.	Lower adherence to the Mediterranean diet was associated with short sleep duration in women.	Gupta et al, 2022 [16]

United Arab Emirates; cross-sectional	503 (MF); 22.1 ± 4.2 yrs	KIDMED	Sleep quality (self-rated sleep quality, sleep latency, sleep duration, sleep efficiency, sleep disturbances, use of sleep medication, and daytime dysfunction).	PSQI	Adherence to the Mediterranean diet was not associated with overall PSQI, but was positively associated with subjective sleep quality, sleep latency, sleep disturbance, and daytime dysfunction.	Naja et al, 2022 [17]

aMED: Alternate Mediterranean Diet score; KIDMED: Mediterranean Diet Quality Index for children and adolescent; MEDAS: Mediterranean Diet Adherence Score; MEDI-LITE: Mediterranean diet adherence score based on the literature; MF: male and female; PDSS: Pediatric Daytime Sleepiness Scale; PSQI: Pittsburg sleep quality index; WHIIRS: Women's Health Insomnia Rating Scale; y: year; yrs: years.

References

1. Jaussent, I.; Dauvilliers, Y.; Ancelin, M.L.; Dartigues, J.F.; Tavernier, B.; Touchon, J.; Ritchie, K.; Besset, A. Insomnia symptoms in older adults: associated factors and gender differences. *Am J Geriatr Psychiatry* **2011**, *19*, 88-97, doi:10.1097/JGP.0b013e3181e049b6.
2. Ferranti, R.; Marventano, S.; Castellano, S.; Giogianni, G.; Nolfo, F.; Rametta, S.; Matalone, M.; Mistretta, A. Sleep quality and duration is related with diet and obesity in young adolescent living in Sicily, Southern Italy. *Sleep Sci* **2016**, *9*, 117-122, doi:10.1016/j.slsci.2016.04.003.
3. Campanini, M.Z.; Guallar-Castillon, P.; Rodriguez-Artalejo, F.; Lopez-Garcia, E. Mediterranean Diet and Changes in Sleep Duration and Indicators of Sleep Quality in Older Adults. *Sleep* **2017**, *40*, doi:10.1093/sleep/zsw083.
4. Arriscado, D.; Knox, E.; Zabala, M.; Zurita-Ortega, F.; Dalmau, J.M.; Muros, J.J. Different healthy habits between northern and southern Spanish school children. *Z Gesundh Wiss* **2017**, *25*, 653-660, doi:10.1007/s10389-017-0823-2.
5. Castro-Diehl, C.; Wood, A.C.; Redline, S.; Reid, M.; Johnson, D.A.; Maras, J.E.; Jacobs, D.R., Jr.; Shea, S.; Crawford, A.; St-Onge, M.P. Mediterranean diet pattern and sleep duration and insomnia symptoms in the Multi-Ethnic Study of Atherosclerosis. *Sleep* **2018**, *41*, doi:10.1093/sleep/zsy158.
6. Mamalaki, E.; Anastasiou, C.A.; Ntanasi, E.; Tsapanou, A.; Kosmidis, M.H.; Dardiotis, E.; Hadjigeorgiou, G.M.; Sakka, P.; Scarmeas, N.; Yannakoulia, M. Associations between the mediterranean diet and sleep in older adults: Results from the hellenic longitudinal investigation of aging and diet study. *Geriatr Gerontol Int* **2018**, *18*, 1543-1548, doi:10.1111/ggi.13521.
7. O'Connor, L.E.; Biberstine, S.L.; Paddon-Jones, D.; Schwichtenberg, A.J.; Campbell, W.W. Adopting a Mediterranean-Style Eating Pattern with Different Amounts of Lean Unprocessed Red Meat Does Not Influence Short-Term Subjective Personal Well-Being in Adults with Overweight or Obesity. *J Nutr* **2018**, *148*, 1917-1923, doi:10.1093/jn/nxy235.
8. Godos, J.; Ferri, R.; Caraci, F.; Cosentino, F.I.I.; Castellano, S.; Galvano, F.; Grosso, G. Adherence to the Mediterranean Diet is Associated with Better Sleep Quality in Italian Adults. *Nutrients* **2019**, *11*, doi:10.3390/nu11050976.
9. Adelantado-Renau, M.; Beltran-Valls, M.R.; Esteban-Cornejo, I.; Martinez-Vizcaino, V.; Santaliestra-Pasias, A.M.; Moliner-Urdiales, D. The influence of adherence to the Mediterranean diet on academic performance is mediated by sleep quality in adolescents. *Acta Paediatr* **2019**, *108*, 339-346, doi:10.1111/apa.14472.
10. van Egmond, L.; Tan, X.; Sjogren, P.; Cederholm, T.; Benedict, C. Association between Healthy Dietary Patterns and Self-Reported Sleep Disturbances in Older Men: The ULSAM Study. *Nutrients* **2019**, *11*, doi:10.3390/nu11051029.
11. Muscogiuri, G.; Barrea, L.; Aprano, S.; Framondi, L.; Di Matteo, R.; Laudisio, D.; Pugliese, G.; Savastano, S.; Colao, A.; on behalf of the, O.P.P. Sleep Quality in Obesity: Does Adherence to the Mediterranean Diet Matter? *Nutrients* **2020**, *12*, doi:10.3390/nu12051364.
12. Zuraikat, F.M.; Makarem, N.; St-Onge, M.P.; Xi, H.; Akkapeddi, A.; Aggarwal, B. A Mediterranean Dietary Pattern Predicts Better Sleep Quality in US Women from the American Heart Association Go Red for Women Strategically Focused Research Network. *Nutrients* **2020**, *12*, doi:10.3390/nu12092830.
13. Flor-Alemany, M.; Nestares, T.; Alemany-Arrebola, I.; Marin-Jimenez, N.; Borges-Cosic, M.; Aparicio, V.A. Influence of Dietary Habits and Mediterranean Diet Adherence on Sleep Quality during Pregnancy. The GESTAFIT Project. *Nutrients* **2020**, *12*, doi:10.3390/nu12113569.
14. Rosi, A.; Giopp, F.; Milioli, G.; Melegari, G.; Goldoni, M.; Parrino, L.; Scazzina, F. Weight Status, Adherence to the Mediterranean Diet, Physical Activity Level, and Sleep Behavior of Italian Junior High School Adolescents. *Nutrients* **2020**, *12*, doi:10.3390/nu12020478.
15. Zaidalkilani, A.T.; Alhaj, O.A.; Serag El-Dine, M.F.; Fekih-Romdhane, F.; AlRasheed, M.M.; Jahrami, H.A.; Bragazzi, N.L. Arab Women Adherence to the Mediterranean Diet and Insomnia. *Medicina (Kaunas)* **2021**, *58*, doi:10.3390/medicina58010017.
16. Gupta, K.; Jansen, E.C.; Campos, H.; Baylin, A. Associations between sleep duration and Mediterranean diet score in Costa Rican adults. *Appetite* **2022**, *170*, 105881, doi:10.1016/j.appet.2021.105881.
17. Naja, F.; Hasan, H.; Khadem, S.H.; Buanq, M.A.; Al-Mulla, H.K.; Aljassmi, A.K.; Faris, M.E. Adherence to the Mediterranean Diet and Its Association With Sleep Quality and Chronotype Among Youth: A Cross-Sectional Study. *Front Nutr* **2021**, *8*, 805955, doi:10.3389/fnut.2021.805955.
